# Centor scores associated poorly with rapid antigen test findings in children with sore throat

**DOI:** 10.1007/s00431-024-05863-2

**Published:** 2024-11-11

**Authors:** Johanna Jääskeläinen, Marjo Renko, Ilari Kuitunen

**Affiliations:** 1 Institute of Clinical Medicine and Department of Pediatrics, University of Eastern Finland, Yliopistonranta 2, 70211 Kuopio, Finland; 2 Department of Pediatrics, Kuopio University Hospital, Kuopio, Finland; 3 Department of Pediatrics, Mikkeli Central Hospital, Mikkeli, Finland

**Keywords:** Group A streptococcus, Pharyngitis, Centor score, Rapid antigen test

## Abstract

The Finnish Current Care Guideline recommends rapid antigen tests as the primary diagnostic tool for both adults and children with Centor score ≥ 3. We aimed to analyze the association of Centor score and rapid antigen test positivity of group A streptococcal pharyngitis (GAS) in Finnish children. We performed a retrospective single-center study from July 2019 to June 2022. We included all children aged 0–15 years based on ICD-10 diagnostic codes for acute pharyngitis. We manually extracted the data from the electronic healthcare records. We extracted the information on Centor score signs and symptoms, rapid antigen tests, throat cultures, and C-reactive protein (CRP) levels. Comparisons were made between different groups by calculating a difference of two proportions with 95% confidence intervals. A total of 464 children were included and rapid antigen tests were taken from 433 (93.3%). We did not detect any significant association between rapid antigen test positivity and Centor scores. Sensitivity of Centor score ≥ 3 for rapid antigen test positivity was 22.3 (95% confidence interval 17.3–27.9) and specificity 79.0% (72.4–84.8). Positive throat culture was found in 17.1% of the patients with negative rapid antigen test. Centor scores correlated positively with CRP levels, but elevated CRP did not predict positive antigen test results. *Conclusion*: The Centor score alone does not seem to be of any utility in guiding the diagnosis of suspected streptococcal pharyngitis. Microbiological testing remains necessary for accurate diagnosis and CRP should not be used to differentiate viral and bacterial pharyngitis cases.

**Table:** 

**What is Known:**
*• The Centor score is a clinical prediction model for differentiating Group A streptococcal (GAS) pharyngitis from viral pharyngitis.*
*• Finnish Current Care Guideline recommends rapid antigen testing in patients with Centor score* ≥ *3 for diagnosing GAS infections and antibiotics prescribed based on confirmed test results.*
**What is New:**
*• The Centor score alone does not seem to have any use in guiding the diagnosis of suspected streptococcal pharyngitis in children.*
*• Microbiological testing remains necessary for accurate diagnosis in patients with sore throat.*

## Introduction

Acute respiratory tract infections are common in children and the vast majority of them are caused by viruses. Group A streptococcal infections (GAS) account for circa 24–37% of acute pharyngitis cases in children [1–3]. Untreated GAS may lead to suppurative or non-suppurative complications and treatment with antibiotics may shorten the symptomatic period. Diagnostic tools such as clinical prediction models, rapid antigen tests, and throat cultures can be used to differentiate GAS infections from viral ones. Targeting the antibiotic treatment to children with true GAS infection may prevent antibiotic overuse and GAS-related complications [4]. Several clinical scoring systems are used to identify patients with a higher likelihood of GAS, such as McIsaac, FeverPAIN, and Centor score.

Centor score is a widely used clinical prediction model (CPM) in GAS diagnosis [5]. Centor score was created in 1981 and validated by Fine et al. in 2012 in an adult population. One point is given from each clinical parameter: tonsillar exudate, tender anterior cervical lymphadenopathy or lymphadenitis, fever (over 38 °C), and absence of cough, to calculate the Centor score [6, 7]. Another CPM used in GAS diagnosis is the McIsaac score which takes patients’ age into account: patients aged 3–14 years and 45 years or older get one additional point. The Finnish national guideline states that rapid antigen test should be taken as a primary microbiological test when the patient presents 3 or more Centor criteria symptoms or signs and that throat cultures should be used only in cases with prolonged symptoms [8]. Antibiotics appear to have only a limited impact on the duration of symptoms in GAS infections. If suppurative complications appear they can be treated accordingly [9, 10]. More stringent approach to initiating antibiotic treatment should be considered. The Finnish current care guideline prefers the Centor score over McIsaac since GAS carriage is more common in children than in adults. The guideline also recommends to avoid CRP testing in patients with sore throat as it does not influence the treatment decisions [8]. Global consensus has not been made on the diagnostic strategies of GAS and there are substantial differences between guidelines. Pellegrino et al. pointed out 3 strategies in managing sore throat, North American and several European guidelines recommend antibiotics in cases of positive microbiological test, whereas Scottish, English, and Dutch guidelines recommend antibiotic treatment for patients with high clinical scores (Centor > 3, FeverPain > 4) and Canadian guideline recommends delayed antibiotic prescription for children with Centor score > 3 and positive throat cultures [5].

In this study, we aimed to analyze how the Centor score associated with rapid antigen test findings and C reactive protein (CRP) levels in Finnish children with sore throat.

## Methods

In this retrospective single-center cohort study, we collected data from pediatric patients (0–15 years) diagnosed with ICD-10 diagnostic codes J02* (pharyngitis) or J03* (tonsillitis) at Mikkeli Central Hospital’s pediatric emergency department between July 2019 and June 2022. Mikkeli Central Hospital’s pediatric emergency department provides primary and secondary-level emergency care 24/7 and the annual visit rate for pediatric patients is between 5000 and 6000. We aimed to gather altogether 400 patients as we hypothesised that this would give enough children to compare children with Centor score < 3 and ≥ 3.

Data were collected from electronic patient records from July 2019 to June 2022. Only primary visits for each acute pharyngitis episode were included in this study. Data were collected manually from the electronic patient records. The following information was gathered: date of visit, age, sex, fever, sore throat complaint, tonsillar status, cervical lymph nodes, Centor score, and results of rapid antigen, throat culture, and CRP tests. If the Centor scores were not recorded, we calculated them for each patient based on symptoms and signs reported on patient records. The results of rapid antigen tests were compared with findings in throat cultures and the results of CRP tests with rapid antigen test results.

Our main outcome was the rapid antigen test positivity rate in children with Centor score < 3 and ≥ 3. RADT was chosen over throat culture since it is recommended as the first-line test in the Finnish national guideline. Secondary outcomes of interest were the mean CRP levels in the different Centor groups and the proportion of rapid antigen test negative samples with GAS growth in culture.

### Statistics

For continuous outcomes means with standard deviations (SD) were presented and categorized variables were presented as absolute numbers and proportions. For categorized variables, we used difference of two proportions with 95% confidence intervals (95% CIs) for the between-group comparisons as it highlights better the uncertainty in the comparisons than standard statistical testing with *p*-values. For continuous variables, we used a *T*-test, and a *p*-value < 0.05 was considered statistically significant. To evaluate the accuracy of diagnostic tests, we calculated sensitivity, specificity, and predictive values with their 95% confidence intervals. We have reported our findings according to STROBE (Strengthening of Reporting in Observational Studies) guidelines, and the checklist is provided in the supplements.

### Permission and ethics

Study permission for this study was granted by the institutional research committee of Mikkeli Central Hospital. According to Finnish research laws, individual consent to participate was not needed due to retrospective study design. The ethical committee statement was waived for this retrospective study by the Northern-Savonia Healthcare region’s ethical committee.

## Results

A total of 465 children were included in this study. The mean age of the children was 9.0 years (SD ± 4.2) and 233 (50.1%) were boys. The majority of the children (83%) did not have any underlying diseases. The most common underlying diseases were asthma and atopic skin disease (6.2%). Only two patients had initially reported Centor scores in their patient records.

Rapid antigen test was taken from 433/464 (93.3%) of the cases. The use of rapid antigen test was lowest in the Centor 0 group as there it was taken from 81.4%, whereas in the other groups (Centor 1–4), the rates were between 94 and 100% (Table 1). Altogether, 59.1% of the rapid antigen tests taken were positive. The proportion of positive results of the antigen tests taken were nearly equal in all Centor score groups as it varied between 54 and 62% (Table 1).

**Table 1:** Rapid antigen test findings and CRP-levels stratified by the Centor scores

Centor score	N of patients	Rapid antigen test taken, *n* (%)	Positive rapid antigen test, *n* (%)	CRP taken, *n* (%)	CRP levels, mean (SD)			*p*-value*
					All	Rapid test +	Rapid test −	
0	59	48 (81.4%)	27 (56.3%)	40 (67.8%)	12.8 (29.0)	17.1 (39.4)	6.2 (10.3)	0.259
1	151	143 (94.7%)	84 (58.7%)	114 (75.5%)	19.6 (28.4)	20.7 (27.0)	19.4 (31.2)	0.799
2	158	148 (93.7%)	88 (59.5%)	126 (79.7%)	32.1 (38.8)	30.8 (33.6)	31.1 (42.1)	0.963
3	85	83 (97.6%)	51 (61.4%)	70 (82.4%)	48.8 (50.7)	38.5 (49.2)	52.1 (53.7)	0.277
4	11	11 (100%)	6 (54.5%)	10 (90.9%)	61.6 (56.2)	39.0 (47.9)	84.2 (59.4)	0.222
Total	464	433 (93.3%)	256 (59.1%)	360 (77.6%)	28.9 (39.6)	27.8 (36.8)	30.3 (42.9)	0.568

*p*-value for *T* test in the comparison of CRP levels between rapid – and rapid test +

Centor score ≥ 3 found children with positive rapid antigen test with sensitivity of 22.3% (95% CI 17.3–27.9) and specificity of 79.0% (72.4–84.8) (Table 2). Predictive values were between 41 and 62%. Throat culture was taken from 205/435 (46.7%) of the patients, 23.0% of patients with a positive rapid test result, and 81.6% with a negative one. The result of the culture was positive in 17.1% of patients with a negative rapid test result and in 47.5% with a positive rapid antigen test result.

**Table 2:** Diagnostic test accuracy of Centor score ≥ 3 for finding children with positive StrA rapid antigen test

	StrA rapid Ag positive	StrA rapid Ag negative	
Centor score ≥ 3	57	37	94
Centor score < 3	199	140	339
	256	177	433

Sensitivity (95% CI) 22.3% (17.3–27.9)

Specificity (95% CI) 79.0% (72.4–84.8)

Positive predictive value (95% CI) 60.6% (50.0–70.6)

Negative predictive value (95% CI) 41.3% (36.0–46.7)

Blood sample for measurement of CRP was taken from 360 (77.4%) of the patients. There was a trend that higher Centor scores were associated with a higher likelihood of measuring CRP (Table 1). The CRP values tended to be higher in children with higher Centor scores, but they did not differ significantly between the patients with negative or positive rapid antigen test results. In the Centor score 0 group, children with positive rapid antigen had higher CRP levels (Table 1).

## Discussion

In this retrospective study, the Centor scores were not associated with rapid antigen test positivity in Finnish children with sore throat. The Finnish Current Care Guideline in its recent update has recommended the use of the Centor score for predicting streptococcal infections in both children and adults. However, the Centor score was not taken into clinical practise as recommended [11]. The Current Care Guidelines also advocate for the use of rapid testing as the primary microbiological test and to avoid unnecessary cultures. Thus, it was surprising to see that over 80% of the samples with negative rapid tests were sent for culturing and that over 20% of the positive samples were also cultured. About 17% of the samples with negative rapid test appeared positive in the culture, the traditional golden standard.

In our study, the highest proportion of positive antigen test results was observed in children with the Centor score 3 and the lowest with the Centor score 4, but the total number of patients was low (*n* = 11) in the Centor score 4 group. Because of the small sample size in Centor score 4 group, the results should be interpreted with caution, although the results are consistent with previous studies. No significant differences in the proportions of positive rapid test results were observed between the Centor score groups. Roggen et al. did a similar study in 2013 in Belgium, and the results were comparable to ours. They found that the Centor score was not effective in ruling in or ruling out GAS in children aged 2 to 16 years [12]. Two systematic reviews have been conducted on the use of the Centor score in children, and neither recommended its use for diagnosing (GAS) infection [13, 14]. The modified Centor score does not seem to be any better than Centor score in predicting GAS infections in children [15].

Considering the number of positive RADTs in lower Centor score groups in our series, it seems that the Centor score does not work at all. The considerable prevalence of GAS carriage in children could be one of the reasons for the number of positive RADTs in lower Centor score groups [3, 16]. However, at the moment, there is no practical way to distinguish viral infection and asymptomatic carriage of GAS from real GAS pharyngitis in a child with a sore throat.

Finland has clear guidance on the national level to avoid CRP testing in patients with sore throat as it does not influence the treatment decisions. In our study, however, nearly 80% of the patients had CRP measured. In many Finnish hospitals, including Mikkeli Central Hospital, pediatric nurses are eligible to take point-of-care testing (CRP, rapid-antigen tests) as part of their initial assessment. This highlights that guidelines targeted towards physicians need to be implemented locally and modified to local practice in order to be effective.

When comparing the average CRP levels with Centor scores and rapid antigen test results, higher CRP levels were observed with increasing Centor scores. However, a higher CRP level did not appear to predict a positive result in the antigen test. In children with negative antigen test result, mean CRP values were similar or higher than in those with positive antigen test result, despite the Centor score 0 group. Based on these results, CRP does not seem to differentiate viral and bacterial pharyngitis but merely correlates with the severity of the symptoms.

The main limitation of our study is the retrospective design and the fact that the Centor scores were not collected prospectively. However, one of our aims was to examine the current real-life practices regarding sore throat diagnostics. Furthermore, we were able to calculate the Centor scores based on the patient records. Also, the high number of rapid antigen testing in each Centor group may not result from practitioners ordering testing alone. Nurses have the option to perform a rapid test at their own discretion before the doctor’s assessment, which may affect the number of RATs taken.

Our study shows an example of noncompliance with national guidelines. Based on the results of this study and literature, the Centor score alone should not be used to rule in or out GAS infections in children with sore throat. The Centor score alone does not seem to be of any utility in guiding the diagnosis of suspected streptococcal pharyngitis. Therefore, microbiological testing is recommended in the diagnostic process of sore throat. However, the interpretation of the test result is left to the clinician’s discretion. We do not recommend treating all positive RADT test results, but only those whose symptoms and findings are also consistent with a GAS infection, since asymptomatic GAS carriage must be taken into account. However, additional costs could be avoided by reducing unnecessary CRP testing by adhering to national guidelines.

We have to change the operational models of local units to ensure adherence to care guidelines. Additionally, nurses working in emergency departments, who have the authority to prescribe tests, should be trained in current care guidelines.

## Data Availability

No datasets were generated or analysed during the current study.

## Declarations

### Study permission

This study was approved by the research committee of Mikkeli Central Hospital.
